# Adipocytes enhance tongue cancer progression potentially via IL-6 and extracellular vesicles

**DOI:** 10.1038/s41598-025-24711-3

**Published:** 2025-11-21

**Authors:** Johanna Peltonen, Riia Tiikkaja, Laura Ketomäki, Sane Taivaanlahti, Tiina Kaakkuriniemi, Susanna Kokkonen, Janne Heikkinen, Sanna Palosaari, Janne Väisänen, Jukka Tikanto, Petri Koivunen, Ahmed Al-Samadi, Maija Risteli, Tuula Salo, Pirjo Åström, Krista Juurikka

**Affiliations:** 1 Department of Oral and Maxillofacial Diseases, Clinicum, University of Helsinki, Helsinki, Finland; 2 Translational Immunology Research Program (TRIMM), University of Helsinki, Helsinki, Finland; 3 Research Unit of Population Health, Faculty of Medicine, University of Oulu, Oulu, Finland; 4 Medical Research Centre Oulu, Oulu University Hospital and University of Oulu, Oulu, Finland; 5 Research Unit of Translational Medicine, Faculty of Medicine, University of Oulu, Oulu, Finland; 6 Department of Otorhinolaryngology, Head and Neck Surgery, Oulu University Hospital, Oulu, Finland; 7 Institute of Dentistry, School of Medicine, Kuopio Campus, University of Eastern Finland, Kuopio, Finland; 8 HUSLAB, Department of Pathology, Helsinki University Central Hospital, University of Helsinki, Helsinki, Finland; 9 Research Unit of Biomedicine and Internal Medicine, Faculty of Medicine, University of Oulu, Aapistie 5A, Oulu, FI-90220 Finland; 10 Biocenter Oulu, Oulu, Finland; 11 Faculty of Biochemistry and Molecular Medicine, University of Oulu, Oulu, Finland

**Keywords:** Adipocytes, Oral tongue cancer, Interleukin-6, Extracellular vesicles, Cancer microenvironment, Oral cancer

## Abstract

Obesity is associated with the increased incidence of various cancers. However, the connection between high body mass index and oral cancer risk is partly controversial, and the mechanistic role of adipocytes is largely unknown. We aimed to study the prognostic value and function of adipose tissue in oral tongue squamous cell carcinoma (OTSCC). The amount of adipocytes in OTSCC samples correlated positively with tumour size. High tumoural inflammatory cell count predicted better overall survival. Patient-derived adipose tissue and adipocytes (differentiated from adipose tissue-derived mesenchymal stem cells) induced the proliferation of OTSCC cells in vitro. Moreover, adipocytes increased the migration of cancer cells from both primary and metastatic sites without the need for direct cell-cell contact. Various cytokines, including interleukin 6 (IL-6), were present at high levels in adipocyte-OTSCC co-cultures, and inhibition of IL-6 signalling markedly reduced cancer cell migration. Moreover, the adipocyte-derived extracellular vesicles (EVs) induced OTSCC cell invasion. We conclude that adipocytes increase the proliferation of cancer cells and enhance their motility without direct cell contact. The protumourigenic effect of adipocytes is likely mediated by secreted cytokines, such as IL-6, and transported via EVs.

**Supplementary Information:**

The online version contains supplementary material available at 10.1038/s41598-025-24711-3.

## Introduction

Obesity is associated with elevated incidence of various cancers such as breast and prostate cancers^1,2^. High body mass index (BMI) is associated with faster cancer progression and worse prognosis^3–6^. As increased body weight is becoming a global health crisis, the role of adipose tissue in cancer cell behaviour is of great interest. The association of higher body weight with the risk of developing oral cancer is partly controversial^7^. Overweight patients with early-stage tongue cancer (T1, T2) have a fivefold higher risk of death than their peers with normal body weight^8^. Similarly, another study that excluded oral tongue squamous cell carcinoma (OTSCC) patients with weight loss before cancer diagnosis found that being overweight doubles the risk of death^9^. In line with this, Iyengar et al.^8^ reported that obesity is an independent predictor of higher risk of death. Moreover, they found that white adipose tissue inflammation locally in the tongue correlates with worse survival^10^. In general, elevated BMI has been reported to be associated with increased fat content in the tongue^11^.

A total of 389 485 new oral cancer cases (including the lip) were diagnosed worldwide in 2022^12^. Of oral cancer cases, 90% are squamous cell carcinomas (SCCs)^13^, and most of them are located in the tongue^14^. Tongue cancers are highly aggressive malignancies that metastasize at an early stage and have poor survival rates (61%) even at early stages I-II^15^. As the cancer progresses from the oral tongue epithelium, the SCC cells invade through the muscular structures to the deeper tissue layers and come into closer contact with adipose tissue^16^.

Adipose tissue acts as an endocrine organ actively participating in metabolic processes and other cellular functions by secreting adipokines (e.g., leptin) and cytokines (e.g., IL-6, IL-8)^3^. Furthermore, obesity enhances the release of these inflammatory mediators from adipocytes and adipose tissue^17^. For example, IL-6 secreted by adipocytes has been shown to be crucial in increasing cancer aggressiveness^18,19^. In addition to being freely secreted, various signalling molecules are transported via extracellular vesicles (EVs). EVs are particles with a diameter of 100–1000 nm^20^, enclosed in a lipid bilayer^21^. They carry, for example RNA, proteins, and metabolites^21^ and are secreted virtually by all cell types^22^, including adipocytes. Obesity further increases the secretion of EVs from adipocytes^23^. Previous studies have shown that EVs from adipocytes enhance the migration and invasion of melanoma cells^24^ and increase proliferation and migration of breast cancer cells^25^. Although crosstalk between oral cancer cells and adipose tissue-derived stem cells has been proposed^26^, the effect of adipocytes and their secreted factors, such as interleukins (ILs) and EVs, on oral cancer remains unclear.

Adipocytes play an important role in metastatic progression, as demonstrated by prostate, ovarian, breast, colon, and skin cancers, which grow and metastasize to adipose-rich environments^3^. Cancer cells can exploit the adipocytes’ energy reservoirs and respond to various secreted signalling molecules^17,18,27^. Pascual et al.^28^ have shown that in oral cancer, particularly the cells initiating the metastases utilize dietary lipids to promote metastasis and express high levels of fatty acid receptor CD36. Oral squamous cell carcinoma (OSCC), including OTSCC, aggressively spreads into neck lymph nodes, and from there the tumour mass penetrates through the lymph node capsule towards the surrounding adipocyte-rich visceral tissue. Furthermore, this extranodal extension is associated with worse prognosis of OTSCC patients^29,30^. However, it is not known how cancer cells respond to adipocyte-derived factors once the metastases have formed.

Both primary and metastatic tumour environments of OTSCC include adipocytes, but their effects on tongue cancer cells’ behaviour are still largely unknown. We aimed to enhance understanding of the functional effects of adipose tissue on both primary and metastatic OTSCC and the impact of adipocyte-derived factors, like IL-6 and EVs, on OTSCC cells. Resolving the molecular crosstalk between these two tissue types can pave the way for novel solutions for targeted treatment of tongue cancer in the future.

## Results

### High tumour-adjacent immune cell infiltration predicts better survival of OTSCC patients

**Fig. 1:**
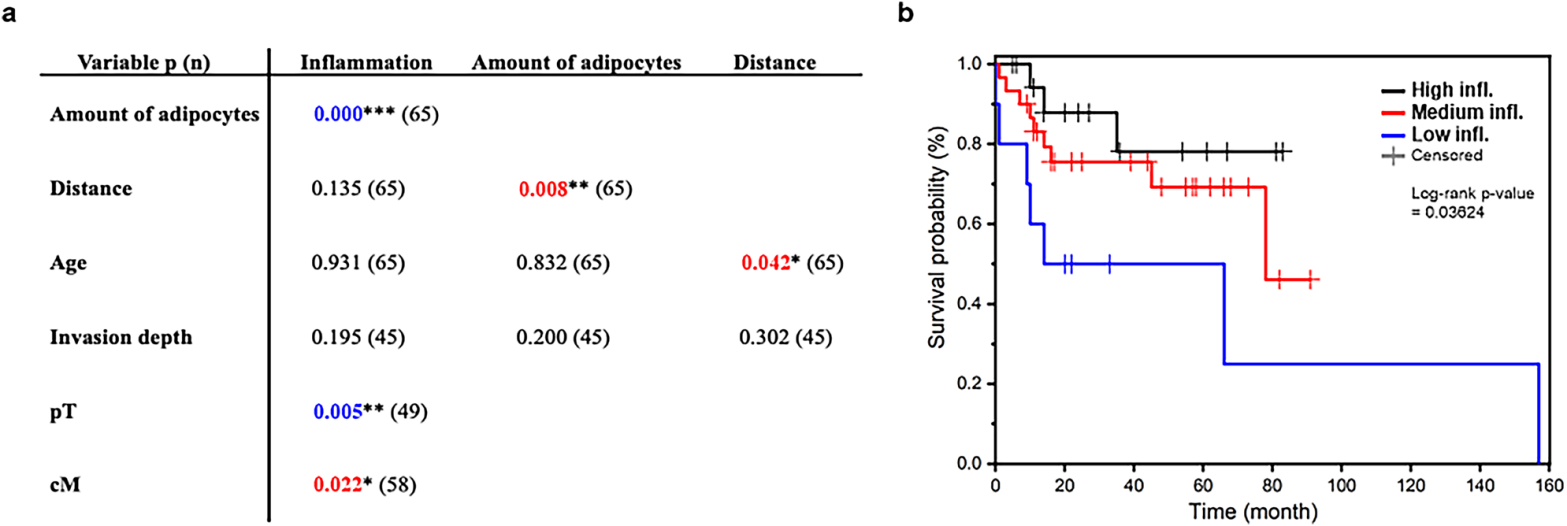
High tumour-adjacent immune cell infiltration predicts better survival of OTSCC patients. Correlation between clinical and measured variables in OTSCC patients assessed with Pearson’s correlation test, Kruskal-Wallis test, Chi-square test, or Fisher’s exact test according to parameter type and normality of distribution **(a)**. Kaplan-Meier survival curve for OTSCC patients grouped by amount of immune cell infiltration (inflammation) at the tumour-adipocyte interface **(b)**. Colour indicates direction of the relationship, blue = positive correlation, and red = negative correlation. Asterisks indicate statistical significance, *p < 0.05, **p < 0.01, and ***p < 0.001. pT = pathological tumour grade, cM = clinical status of distant metastasis.

In human OTSCC tumours, the distance between cancer cells and adipocytes correlated with the number of adipocytes in the sample (*p* = 0.008) and the age of the patient (*p* = 0.042) (Fig. 1a). Moreover, high tumour-adjacent immune cell infiltration in the tumour-adipose tissue interface correlated with better overall survival (*p* = 0.036) of the patients (Fig. 1b). Interestingly, high immune cell infiltration correlated with increased tumour size (pT, *p* = 0.005) and a greater number of adipocytes (*p* < 0.001) (Fig. 1a). To conclude, we determined that adipose tissue is a prominent feature of tumor microenvironment (TME) in OTSCC tumors.

### Adipose tissue and adipocytes show tumor-promoting effects on OTSCC cells

**Fig. 2:**
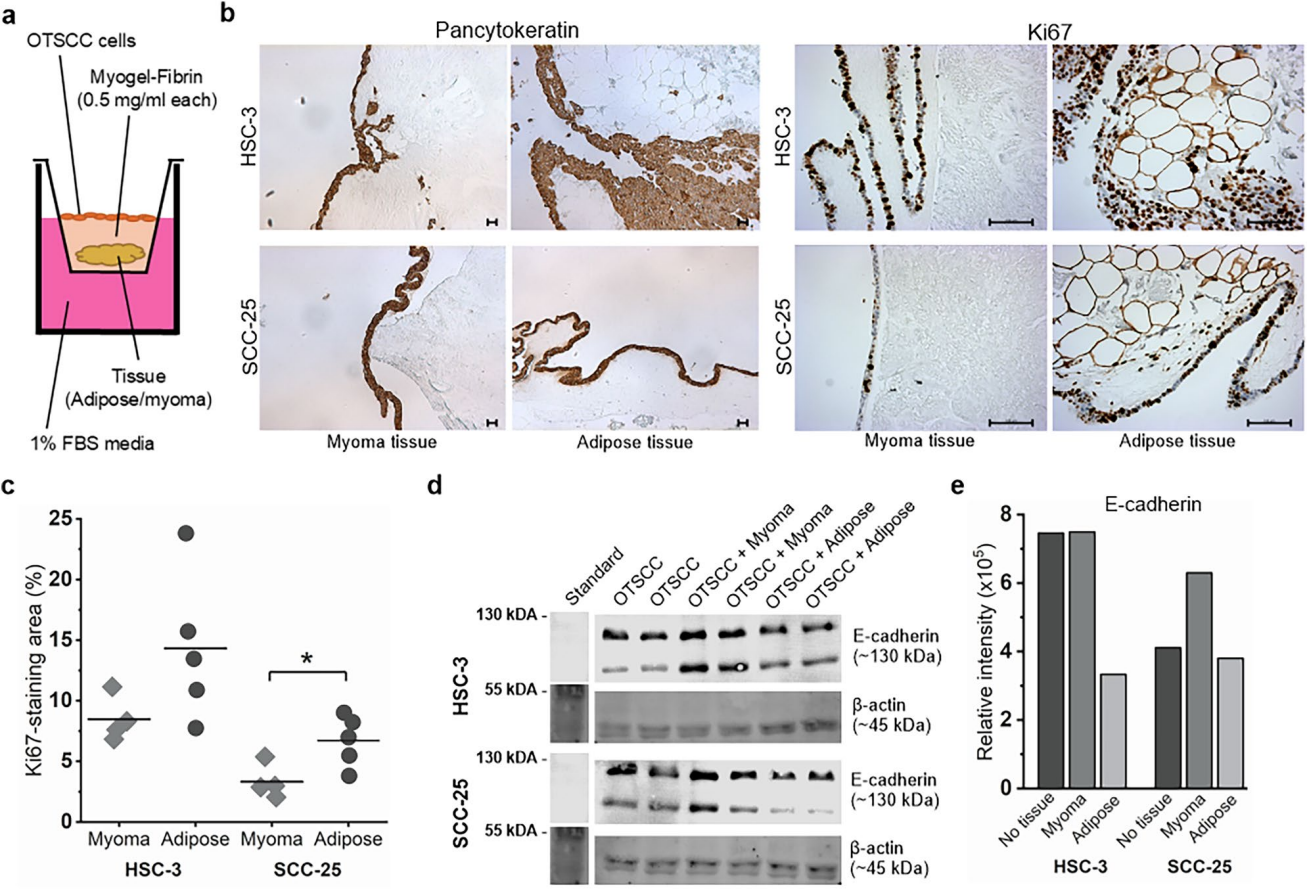
OTSCC patient-derived adipose tissue (pdAT) enhances OTSCC cell proliferation and EMT. Schematic overview of the co-culture conditions **(a)**. Pancytokeratin and Ki67 staining of OTSCC cells (HSC-3 and SCC-25) grown with patient-derived adipose tissue (pdAT) or myoma tissue control **(b)**. Ki67 staining area (%) in OTSCC cells grown with adipose/myoma tissues **(c)**. Representative immunoblot (**d**) and quantification (**e**, average of two replicates presented) of E-cadherin in different co-culture conditions. Scalebar represents 100 μm, 10x objective used for pancytokeratin and 20x objective for Ki67 evaluation. Asterisks indicate statistical significance as evaluated with Student’s T-test, *p < 0.05.

As adipose tissue was present in OTSCC TME in our patient cohort, we wanted to determine how adipose tissue and adipocytes affect OTSCC cell behaviour in vitro. Indeed, we saw an increase in proliferation and induction of EMT when OTSCC cells were cultured with adipose tissue compared to TME mimicking tissue control, myoma. Ki67 staining of OTSCC cells co-cultured in vitro with patient-derived adipose tissue (pdAT) showed enhanced cancer cell proliferation compared with myoma tissue used as a non-adipocyte-rich control tissue. When cultured with adipose tissue (Fig. 2a), there were more cancer cells (nuclei/counts, *p* = 0.018 for HSC-3 and *p* = 0.051 for SCC-25), the cell mass covered a larger total area (*p* = 0.084 for HSC-3, *p* = 0.037 for SCC-25), and a higher percentage of cells were dividing (% of total area, *p* = 0.110 for HSC-3, *p* = 0.029 for SCC-25) (Fig. 2b-c). As loss of E-cadherin, a marker of epithelial–mesenchymal transition (EMT), has been associated with an increase in (OSCC) tumour cell proliferation in mice studies^31^ and malignant transformation in oral cavity lesions in human samples^32^, we examined whether co-culture with adipocytes affects E-cadherin levels in OTSCC cells. Co-culture with adipose tissue decreased E-cadherin in OTSCC cells compared with myoma tissue or no tissue. This decrease was seen more promptly in HSC-3 cells than in SCC-25 cells (Fig. 2d-e).

**Fig. 3:**
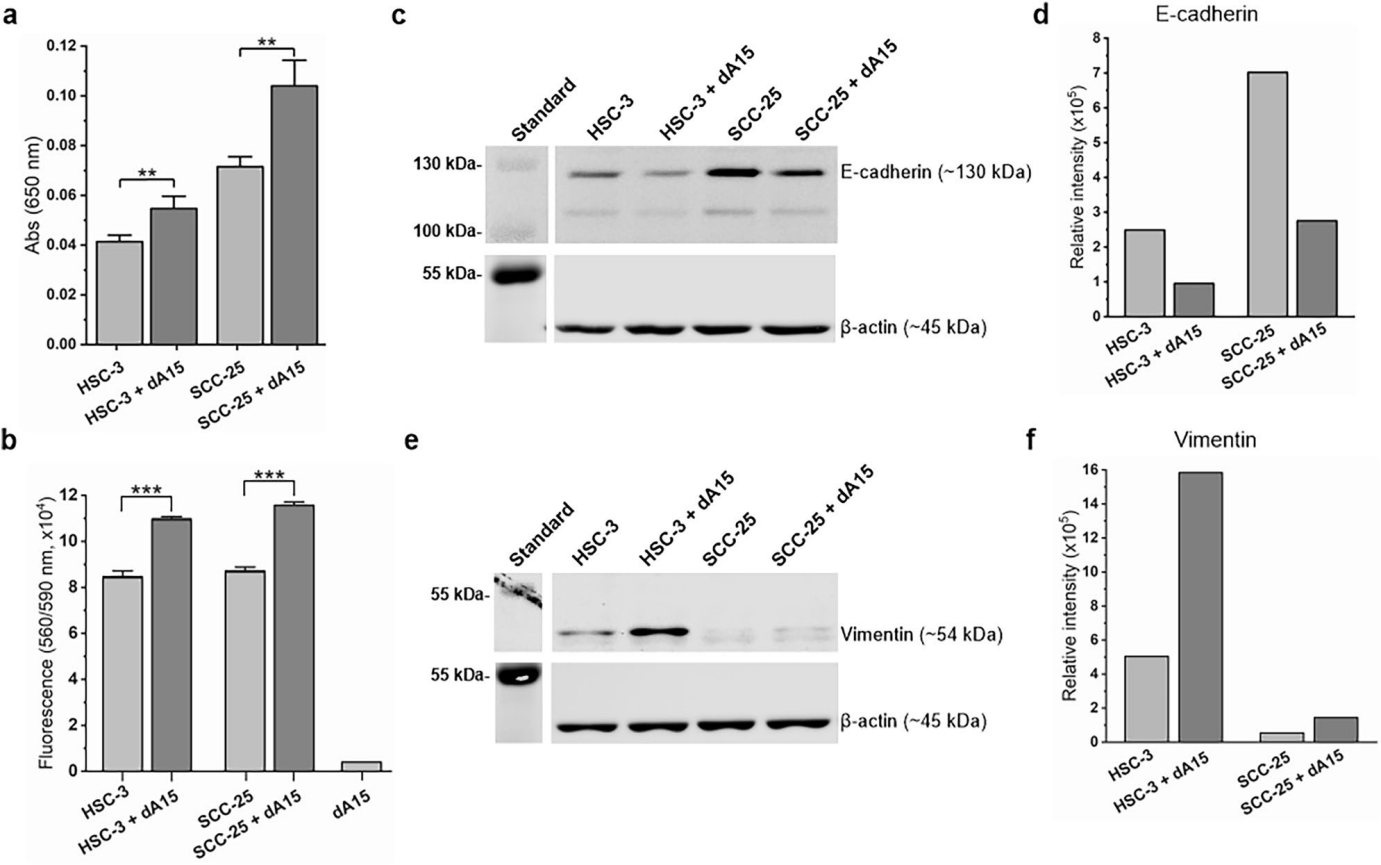
Adipocytes enhance OTSCC migration and viability. OTSCC cells (HSC-3 and SCC-25) were cultured with and without differentiated adipocytes (dA15). OTSCC cell migration was studied with Transwell migration assay **(a).** Resazurin assay was used to assess OTSCC viability **(b)**. Representative immunoblots (**c**,** e**) and quantifications (**d**,** f**) of EMT markers, E-cadherin (**c**,** d**), and vimentin (**e**,** f**). Asterisks indicate statistical significance as evaluated with Student’s T-test, ***p < 0.001.

Next, we wanted to further validate our findings on cell level using adipocytes differentiated from mesenchymal stem cells. Adipocytes increased the migration of HSC-3 (*p* = 0.0032) (Fig. 3a), SCC-25 (*p* = 0.0011) (Fig. 3a), UT-SCC-24 A (*p* = 0.025) (Supplementary Fig. 1a), UT-SCC-42 A (*p* = 0.002) (Supplementary Fig. 1b), and UT-SCC-42B (*p* = 0.001) (Supplementary Fig. 1b) in Transwell migration assay. Additionally, an increase in viability was detected when HSC-3 (*p* < 0.001) and SCC-25 (*p* < 0.001) were co-cultured with adipocytes (Fig. 3b). Yet, active interaction (without direct physical connection) between cancer cells and adipocytes seems necessary, as no changes in migration was seen when OTSCC cells were removed from co-cultures with adipocytes (Supplementary Fig. 2). Migration is a crucial phenomenon in the metastatic cascade and cancer progression. Since adipocytes increased OTSCC cell migration, we decided to study the effect of adipocyte co-culture on the expression of EMT markers in OTSCC cells. Indeed, co-culture with adipocytes decreased E-cadherin, a marker of EMT, in OTSCC cells but more prominently in SCC-25 cells (Fig. 3c-d). An increase in the levels of vimentin, another marker of EMT, was observed when OTSCC cells (especially HSC-3) were co-cultured with adipocytes (Fig. 3e-f). Taken together, our results show that both adipose tissue and adipocytes support OTSCC aggressiveness.

### Adipokine interleukin-6 increases OTSCC cell migration

**Fig. 4:**
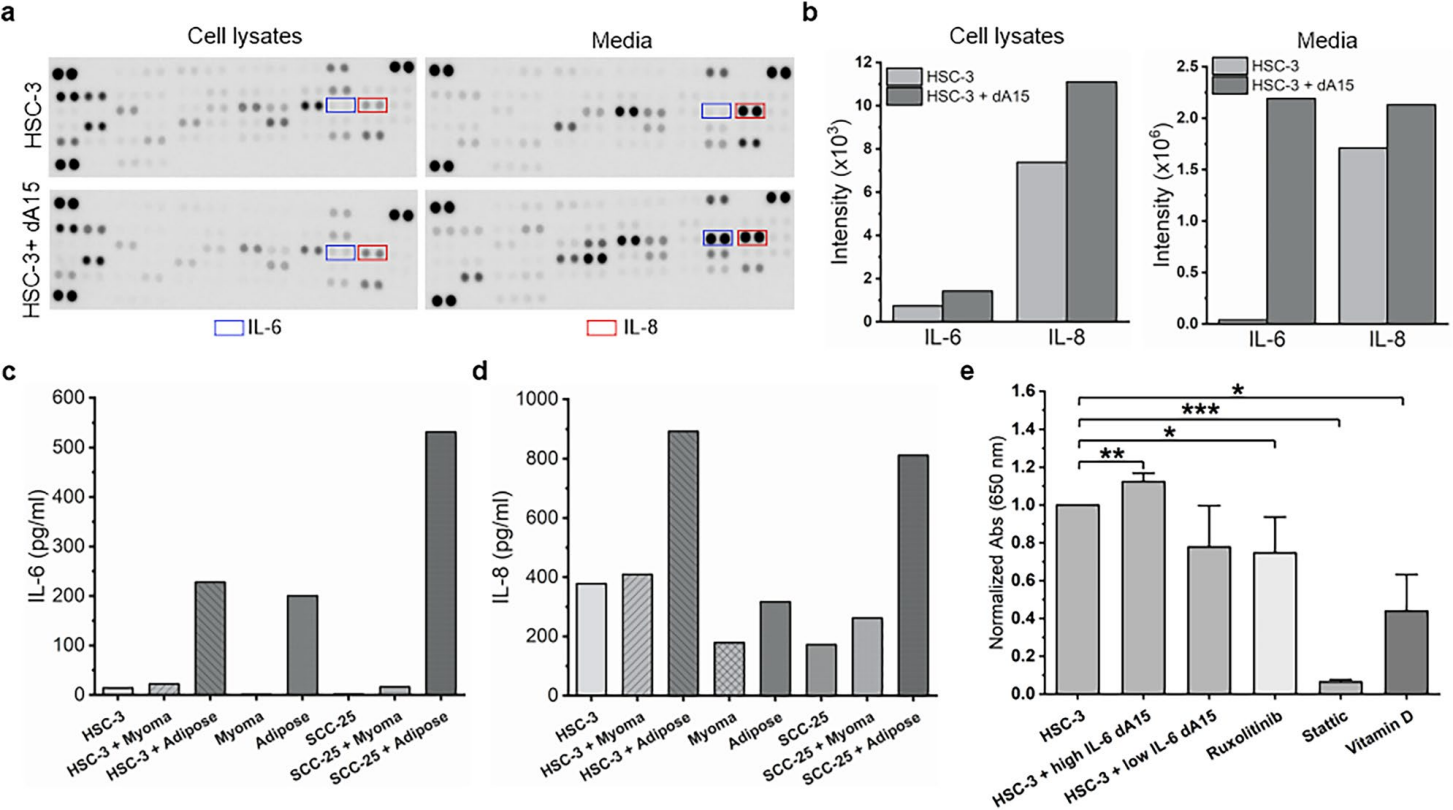
Adipocytes and adipose tissue secrete IL-6, which is required for OTSCC migration. Proteome Profiler human adipokine array kit was used to study the secretomes and cell lysates from HSC-3 cells cultured alone or with adipocytes **(a**,** b)**. Levels of IL-6 and IL-8 were measured from OTSCC cells co-cultured with adipose tissue, myoma tissue, or no tissue with ELISA **(c**,** d)**. The dependency of HSC-3 cell migration on IL-6 was assessed in Transwell migration assay with adipocytes expressing different levels of IL-6 and known IL-6 signalling pathway inhibitors Ruxolitinib, Stattic, and vitamin D **(e)**. Asterisks indicate statistical significance as evaluated with Student’s T-test, *p < 0.05, **p < 0.01, and ***p < 0.001.

Because adipocytes induced OTSCC cell migration even when there was no direct cell-cell contact, we hypothesized that adipocyte-secreted soluble factors were involved in mediating the promigratory effects. Various adipokines, including IL-6 and IL-8, increased in the media and OTSCC cell lysates from co-cultures compared with HSC-3 cells alone (Fig. 4a, Supplementary Fig. 3). IL-6 was especially increased in media from HSC-3 cells co-cultured with adipocytes, whereas increased IL-8 was more prominent in cell lysates (Fig. 4b).

We further studied IL-6 and IL-8 levels in OTSCC co-cultured with adipose tissue, control tissue (myoma), or no tissue with ELISA. Co-culture of HSC-3 and SCC-25 cells with patient-derived adipose tissue showed higher levels of IL-6 (Fig. 4c) and IL-8 (Fig. 4d) than co-culture with control tissue (myoma) or without any tissue. Interestingly, we also noted that adipocytes secrete different levels of IL-6, leading to differing effects; high IL-6 secretion strongly induces OTSCC migration (*p* = 0.006), contrary to adipocytes with low IL-6 secretion (Fig. 4e). Furthermore, the migration of HSC-3 cells was lower when cultured with inhibitors affecting IL-6 signalling (Ruxolitinib, *p* = 0.0455, Stattic, *p* = 0.00004, and vitamin D, *p* = 0.0144) (Fig. 4e), further implicating the promigratory effect of IL-6 signalling in OTSCC cells.

### Adipocyte-derived extracellular vesicles induce OTSCC cell invasion

**Fig. 5:**
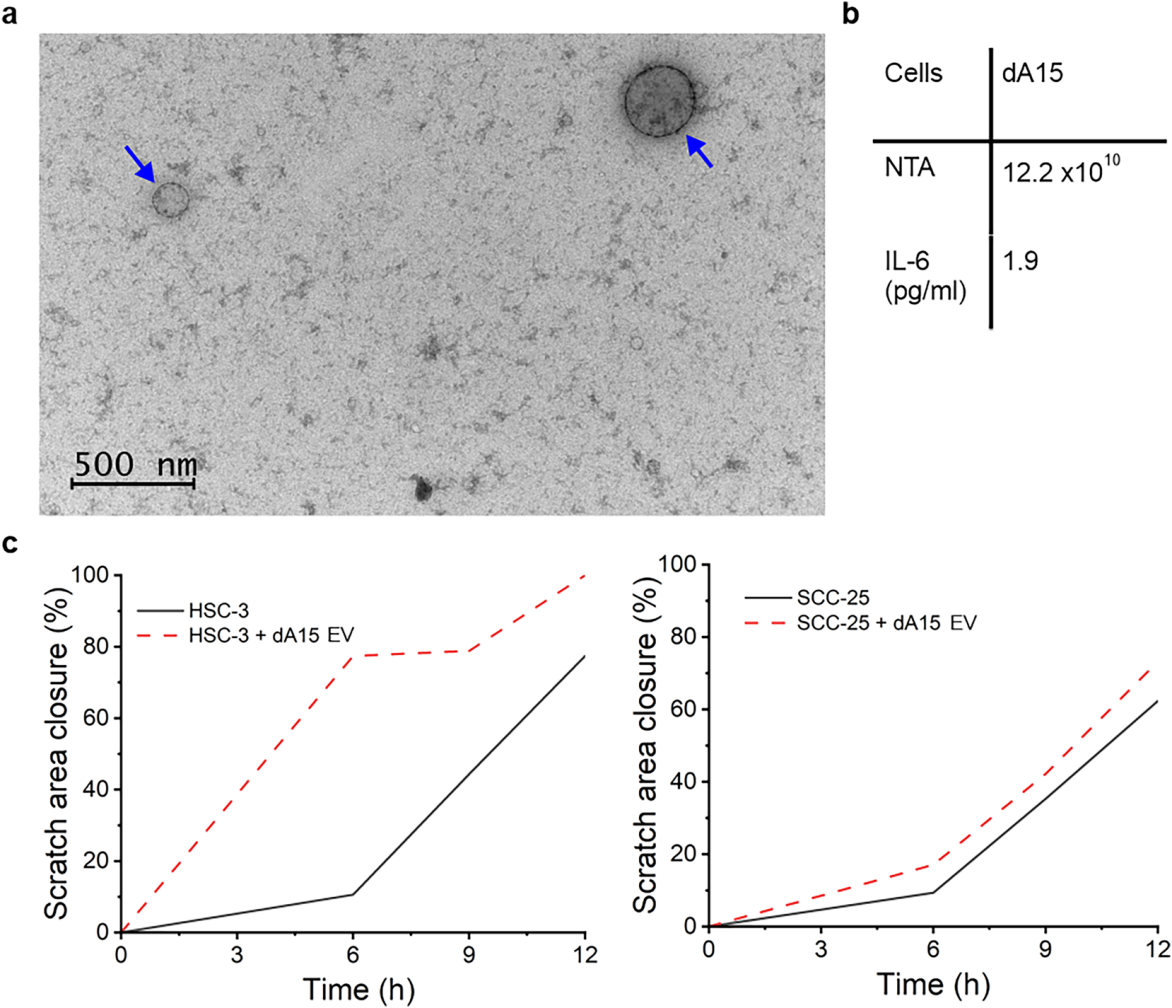
Adipocytes secrete extracellular vesicles (EVs), which enhance OTSCC motility. EVs were characterized by immunoelectron microscopy (IEM) **(a)**. Cargo analysis was performed with ELISA to analyse IL-6 levels of EVs from differentiated adipocytes (dA15) **(b).** EVs’ effect on OTSCC cell invasion was measured by scratch wound assay in Incucyte **(c)**. Blue arrows indicate EVs and scalebar represents 500 μm in IEM analysis. NTA = Nanoparticle tracking analysis (particle/ml).

Adipose tissue can exert its effects locally but also on distant targets by secreting EVs. We demonstrated that adipocytes secrete EVs in culture conditions, as detected by immunoelectron microscopy (Fig. 5a), with a concentration of 12 × 10^10^ particles/ml (Fig. 5b). These adipocyte-derived EVs increased OTSCC cell invasion in an Incucyte wound healing assay (Fig. 5c). The EVs were also shown to contain IL-6, which could, along with other factors that EVs carry, participate in increasing the invasion of OTSCC cells (Fig. 5b). Based on our results, adipose tissue and adipocytes enhance OTSCC aggressiveness by secreting soluble factors, that could be transported via EVs.

## Discussion

Obesity is a rising health problem worldwide and is known to increase incidence and progression of various cancers^1^. However, research on the biological effects of adipocytes in obesity and tongue cancer remains limited. Our aim was to elucidate the connection between obesity and tongue cancer using patient samples and in vitro co-culture models.

To study the potential connection of adipose tissue with OTSCC, we examined patients’ primary tumour tissue histological sections. These analyses showed that a reduced distance between cancer cells and adipocytes correlated with a high number of adipocytes in the sample and age of the patient. Interestingly, high immune cell infiltration in the tumour-invasive front predicted better survival of OTSCC patients in our study. Similar observation have been made by others before^33,34^ and our finding strengthens the idea of immune cell infiltration as a marker for better prognosis in OTSCC patients. Previous studies have shown that higher body weight and the subsequent increase in the amount of adipose tissue lead to adipose tissue hypoxia, adipocyte hypertrophy, and hyperplasia. This results in low-grade inflammation in adipose tissue, which alters the microenvironment in a more favourable direction for cancer growth and development^3^. Body mass index (BMI) of 30 kg/m^2^ or more was associated with a fivefold increase in the risk of death from OTSCC. This study showed that tongue white adipose tissue inflammation in patients with OTSCC is associated with adverse pathological features, including increased tumour thickness and vascular invasion^10^. Survival analysis of OSCC patients revealed that poor prognosis was associated with increased quantity of adipocytes and high expression of CD33 (marker for myeloid-derived suppressor cells, which facilitate tumour growth)^35^.

Neck adipose tissue from patients with head and neck squamous cell carcinoma (HNSCC) co-cultured in contact with OTSCC cells in vitro induced proliferation of cancer cells more than leiomyoma tissue (benign tumor) used as a control tissue, as demonstrated by Ki67 staining. Human myoma tissue has been shown to perform better in invasion assays than healthy porcine tongue tissue^36^, thus it adequately mimics tumour microenvironment and was considered a relevant control tissue in our study. Because of our findings from patient samples and the observed effects of adipose tissue on OTSCC cells’ proliferation, we wanted to further investigate the effects of adipose tissue and adipocytes on tongue cancer cells. We found that adipocytes differentiated from adipose tissue-derived mesenchymal stem cells increased tongue cancer cells’ migration and proliferation. Previous findings of the effect of adipose-derived stem cells (ADSCs) on OTSCC cells are partly conflicting. A study^37^ using co-culture of OTSCC cells and ADSCs did not report changes in OTSCC (HSC-3) migration or invasion. Furthermore, conditioned media from ADSCs did not change significantly the gene expression in OTSCC cells. Yet, Li et al.^26^ showed that OTSCC (Cal27) cells cultured with conditioned media from ADSCs migrated more and had activated Wnt-signalling. They also demonstrated that OTSCC cells injected together with ADSCs into nude mice proliferated more than cancer cells injected alone. Furthermore, Peng et al.^35^ showed that a high-fat diet accelerated 4-nitroquinoline-1-oxide (4NQO) -induced oral tongue carcinogenesis in mice. More studies in this area are needed to clarify the connection between adipose tissue, ADSCs, and OTSCC as well as the mechanisms involved in their interactions.

Based on our results, adipocytes influence OTSCC cells’ motility even without direct cell-cell contact. This suggests that adipocytes secrete factors that induce migration of OTSCC cells. Adipocytes are known to secrete ILs^3^, which have varying effects on tumour progression. For example, IL-6 and IL-8 have been shown to promote tumour growth and formation of metastases^38^. Indeed, media from OTSCC cell and adipocyte co-cultures contained high levels of IL-6, which could be one of the factors promoting migration. Iyengar et al.^10^ also suggested that proinflammatory mediators from adipose tissues, such as IL-6, could have an effect on tongue cancer progression. It has been speculated that inflammation of adipose tissue even in distant parts of the body may play a role in head and neck cancers due to systemic effects such as increased circulating IL-6 levels. The protumourigenic role of IL-6 in OSCC was supported by the finding that overexpression of IL-6 in OTSCC cells increased their proliferation in vitro and tumour growth in vivo^39^. In parallel, supplementing OTSCC cells with recombinant human IL-6 or IL-6 production inducing agents led to increased proliferation^40,41^ and migration^42^ and these effects could be reversed by inhibitors of IL-6 signalling (Stattic) or anti-IL-6 antibodies, comparable to our results discussed in next paragraph. In animal experiments, a significant reduction in tumor volume of tongue cancer xenografts was observed when IL-6 receptor was inhibited with a specific antibody (tocilizumab)^43,44^. Interestingly, high IL-6 expression in OSCC tumours was shown to predict worse disease-free survival^39^. Moreover, elevated IL-6 levels in blood were associated with worse overall survival in OSCC patients^45^.

Studies have shown that vitamin D lowers IL-6 production of immune cells, yet similar effects may also occur in other cell types. Here, we showed that migration of HSC-3 cells was inhibited when the cells were cultured with vitamin D or inhibitors of IL-6 signalling, suggesting that the migration of OTSCC cells highly benefits from the presence of IL-6. A recent meta-analysis indicated strong reduction in total cancer mortality and suggested a reduced risk of various cancers (breast, colorectal, lung, renal, and head and neck cancers) after vitamin D3 supplementation^46^. Moreover, current evidence suggests a possible association between vitamin D deficiency and an increased risk of oral cancer^47–49^. However, more studies are required to evaluate the potential of vitamin D in prevention of oral cancer.

In addition to cytokines, EVs play an important role in cell communication. Interestingly, we found that adipocyte-derived EVs induced OTSCC cell invasion. We demonstrated that these EVs contained IL-6, which could therefore be one of the factors mediating the proinvasive effects. According to the literature, circulating EV levels are regarded as potential markers for cancer diagnosis and prognosis and are significantly higher in obese patients^50^. Matilainen et al.^23^ showed that factors associated with adipose tissue inflammation (e.g. TNFα) in obesity induced the secretion of adipose tissue EVs and changed their fatty acid profiles. In line with our findings, previous studies have shown that EVs from adipocytes increase the migration and invasion in various cancer cells^24,25^. It is acknowledged that adipocytes can trigger modifications in distant tumour cells by exchange of EVs through circulation^51^. Our study highlights the fact that obesity might play a significant role also in tongue cancer progression. While it has been shown that obesity is associated with elevated incidence of various cancers^1,2^, studies on the mechanistic effects of adipocytes in tongue cancer have been lacking.

Our findings demonstrate the tumour-promoting effects of adipocytes on tongue cancer cells, suggesting IL-6 and EVs as one of the mediators of these effects. Yet, we must point out that these effects are likely multifactorial. These adipocyte-derived factors could take part in disease progression, particularly in obese tongue cancer patients, and their targeting may be considered in personalized cancer treatments in the future. However, more research is needed to demonstrate the effects of adipocyte-derived IL-6 (for example by conditional knockout of IL-6 production in adipocytes) or inhibition of EV secretion on tongue cancer progression in in vivo animal experiments.

## Materials and methods

### Human samples

Haematoxylin–eosin-stained sections from formalin-fixed, paraffin-embedded archival resection specimens (primary tumour) of OTSCC patients (*n* = 65) treated in 2009–2016 at Oulu University Hospital were used. The use of patient tissues and data was approved by the Ethics Committee of the Northern Ostrobothnia Hospital District (statement nos. 49/2010 and 101/2020) and Fimea (FIMEA/2020/007614 and former Valvira 6865/05.01.00.06/2010). Patient characteristics are described in Supplementary Table 1.

Fresh adipose tissue adjacent to the neck lymph nodes with suspected metastasis was resected during lymph node dissection surgery from the oral tongue cancer patients. The adipose tissue was placed into 1 x phosphate-buffered saline (PBS) containing 200 U/ml penicillin and 200 µg/ml streptomycin (both from Sigma-Aldrich). Tissues were cut into 1–2 mm pieces and immediately used in co-cultures with carcinoma cells as described below. The use of resected adipose tissue was approved by the Ethics Committee of the Northern Ostrobothnia Hospital District (statement no. 31/2016). Uterine leiomyoma tissue (use approved in statement no. 2/2017) served as a control. We chose myoma tissue as a control as it has been validated and used to mimic local tumor microenvironment (TME) in various tumor types (see for example, colorectal cancer^52^, oral cancer cells^53^ and lung cancer^54^). Informed consent was obtained from all subjects, when required. The study was performed in accordance with the Declaration of Helsinki.

### Analysis of adipose tissue regions and inflammation in resected OTSCC samples

The amount of adipocytes (in quartile range), the shortest distance (µm) between adipocytes and the primary tumour, and the presence of inflammatory cells in the adipocyte-rich region (in quartile range) were evaluated by two individuals from the histological sections with a Leica DM4000B microscope and the Leica Application Suite (LAS) V4.6 program at 5x objective.

### Cell culture

Carcinoma cells: the highly aggressive OTSCC cell line HSC-3 (JCRB Cell Bank), originally isolated from a metastatic site, and the SCC-25 cell line (ATCC), originally isolated from a primary tumour site, were used in most experiments. Additionally, primary tumour and metastatic cell line pairs for OTSCC (UT-SCC-24 A and -B) and laryngeal SCC (UT-SCC-42 A and -B) were used in some experiments (a kind gift from Professor Reidar Grénman, University of Turku, Finland). All cells were cultured in Dulbecco’s Modified Eagle medium (DMEM) with Nutrient Mixture F-12 (Gibco), 10% foetal bovine serum (FBS, Gibco), 100 U/ml penicillin, 100 µg/ml streptomycin, 50 µg/ml ascorbic acid, 250 ng/ml amphotericin B, and 0.4 ng/ml hydrocortisone (all from Sigma-Aldrich).

adMSCs: Adipose-derived mesenchymal stem cells (adMSCs) A15 and A16 were a kind gift from Professor Bettina Mannerström (University of Helsinki), and adMSC A15 used in the EV experiment was a kind gift from Professor Riitta Seppänen-Kaijansinkko (University of Helsinki). The adMSCs were cultured in Dulbecco’s Modified Eagle medium (DMEM, Sigma Aldrich) supplemented with 10% heat inactivated foetal bovine serum (FBS), 100 U/ml penicillin, 100 µg/ml streptomycin, 1 mM sodium pyruvate, 50 µg/ml ascorbic acid, and 250 ng/ml fungizone. Differentiation to adipocytes (named dA15 and dA16 after differentiation) was initiated by washing the cells with 1 x PBS and changing to the complete media supplemented with 1 µM dexamethasone, 1µM indometacin, and 500 µM 3-isobutyl-1-methylxanthine (all from Sigma Aldrich) and Insulin-Transferrin-selenium supplement (Gibco). For collection of extracellular vesicles from adipocytes, differentiation was initiated with StemPro™ Adipogenesis Differentiation kit (Gibco). The adMSCs were differentiated for 3 weeks before they were used in experiments. Adipogenicity was assessed with Oil red O staining.

Co-cultures: OTSCC cells were co-cultured with adipocytes differentiated from adMSCs. In co-cultures with tissue pieces, 1 × 10^5^ OTSCC cells per 6 cm Petri dish were plated and the following day tissue pieces were added on the Petri dishes on metal grids with polystyrene membrane – 1–2 pieces of tissue per grid, 2 grids per Petri dish. Myoma tissue and grids without any tissue were used as controls. After 7 days, the tissues were discarded, and the cells were used to extract proteins. To prepare co-culture samples for immunohistochemistry, adipose tissue pieces from OTSCC patients or similarly sized myoma tissue pieces (control) were embedded in Myogel-Fibrin matrix (0.5 mg/ml Myogel, 0.5 mg/ml fibrinogen from Merch Millipore, and 0.3 U/ml thrombin and 33.3 µg/ml approtinin, both from Sigma Alrich) with 1% FBS culture media in a Transwell insert (Corning) with 8 μm pore size. After the matrix had solidified, 7 × 10^4^ HSC-3 or SCC-25 cells were plated on top. After 5 days, the pieces were removed from the Transwell and fixed overnight in 4% paraformaldehyde solution at room temperature (RT).

### Immunohistochemistry

Tissue co-culture samples were cut into 4 μm sections, deparaffinized, and rehydrated (Tissue-Tek^®^DRS™2000). Antigen retrieval was performed in Tris‐EDTA buffer (pH 9). Endogenous peroxidase activity was blocked with Dako REAL™ peroxidase blocking solution (10 min RT, Dako). Mouse anti-pancytokeratin AE1/AE3 antibody (30 min at RT, 1:200, Dako) was used to study the invaded cancer cells. To study OTSCC proliferation, sections were stained (30 min at RT) with mouse monoclonal Ki67 antibody (Ready-to-use, Enzo Life Sciences). EnVision™ Detection Systems (Peroxidase/DAB, mouse/rabbit, K5007, Agilent) was used for visualization. Counterstaining was performed with Mayer’s haematoxylin. The staining was evaluated under a LeicaDM4000B microscope and imaged with a Leica LCF320 camera using Leica Application Suite software using 10x or 20x objective.

### Western blot

Cells were washed once with cold 1 x PBS and scraped on ice to extraction buffer (50 mM Tris-HCl pH 7.5, 10 mM CaCl_2_·H_2_0, 150 mM NaCl, 0.05% Brij35) supplemented with Complete™ Protease Inhibitor Cocktail (Roche Diagnostics) and Halt™ Phosphatase Inhibitor (Thermo Fisher Scientific). After overnight incubation at 4 °C with shaking, the cell debris was removed by centrifugation (10 min, 4 °C, 17000 x g). Total protein was assessed with NanoDrop 2000 (Protein A280, Thermo Fisher Scientific). Proteins (30 µg) were separated by 12% sodium dodecyl sulfate-polyacrylamide gel electrophoresis and transferred to an Immobilon^®^-FL membrane (Millipore) using the Trans-Blot Turbo Transfer system (Bio-Rad Laboratories). Non-specific antibody binding was blocked with the Odyssey^®^ Blocking Buffer (1 h RT, LI-COR Biosciences), after which the membrane was washed with TBS-Tween^®^20 (0.05%, Sigma Aldrich) (TBS-T) and incubated with 1:500 rabbit polyclonal vimentin antibody (NBPI 19480, Novus Biologicals) or 1:2000 rabbit polyclonal E-cadherin antibody (#3195, Cell Signaling Technology) overnight at 4℃. After washing 3 × 5 min with TBS-T, the membrane was incubated with the IRDye^®^ 680RD goat anti-rabbit secondary antibody (1:10000, LI-COR Biosciences) for 40 min at RT. 1:2000 mouse anti-β-actin (ab8226, Abcam, 4 h at RT) was used as loading control and detected with IRDye^®^ 800CW goat anti-mouse secondary antibody (1:10000, LI-COR Biosciences). The Odyssey scanner (LI-COR Biosciences) was used for imaging, and band intensities were quantified using Image StudioLite (LI-COR Biosciences). The obtained values were normalized to the corresponding β-actin level. Full blot images are available in the supplementary information file.

### Isolation and characterization of extracellular vesicles

EV-depleted serum was prepared by centrifugation of the FBS (Gibco) at 100 000 x g for 19 h at 4 °C, and the upper light-coloured layer was collected and filtered through a 0.22 μm filter^55^. EV-depleted serum was stored at -20 °C until further use.

The subconfluent-differentiated adipocytes were cultured in a medium-sized flask (T-75) in DMEM media supplemented with 10% EV-depleted FBS for 48 h. The conditioned media were collected, centrifuged at 300 x g for 2 min, and stored at -80 °C. The EV isolation was performed as previously described^56^. Briefly, after thawing on ice, the conditioned media were centrifuged at 10 000 x g for 90 min at 4 °C. The supernatant was collected in a new tube, and the EVs were pelleted with centrifugation at 100 000 x g for 90 min at 4 °C. The supernatant was discarded, the pellet was suspended in 200 µl PBS, and the EVs were stored at -80 °C until further use.

Characterization of EVs from differentiated adipocytes was performed using nanoparticle tracking analysis (Nanosight model LM14; Nanosight). To identify EV-particles immuno-electron microscopy (IEM) in the EV Core at University of Helsinki was used. Analysis of EVs with immunostaining was performed as described in Puhka et al.^57^ with the addition of an immunostaining step. Briefly, after loading to 200 mesh copper grids, the samples were blocked with 0.5% BSA in 0.1 M NaPO_4_ buffer (pH 7.0) and then incubated with anti-CD9 (1/200 dilution, HBM-CD9) in 0.1% BSA/NaPO_4_ buffer. Next, the samples were incubated with 10 nm gold nanoparticle conjugated goat anti-mouse IgG (1/80 dilution, BBI Solutions) in 0.1% BSA/NaPO_4_ buffer, washed with NaPO_4_ buffer and deionized water, negatively stained with 2% neutral uranyl acetate and embedded in methyl cellulose uranyl acetate mixture (1.8/0.4%). EVs were viewed with transmission EM using Jeol JEM-1400 (Jeol Ltd.) operating at 80 kV. All samples presented EVs of typical morphology and variable sizes, but small vesicles appeared to predominate. Positive staining with CD9 was detected in the EV samples (not shown).

### Proliferation assays

To examine the effect of adipocytes on OTSCC proliferation, 6 × 10^3^ OTSCC cells per well were seeded onto a 24-well plate and 1 × 10^4^ adipocytes were seeded into the Transwell chambers with 8 μm pore size (Corning). Media without cells were used as a control, and all conditions were performed in quadruplicate. After 72 h co-culture, resazurin sodium salt in a final concentration of 3 µg/ml (Sigma-Aldrich) was added to the bottom well. After 3 h incubation at 37 °C, the fluorescence was measured at 560 nm/590 nm (excitation/emission) with a Victor3V (Perkin Elmer) plate reader. The experiment was repeated three times. To examine the effect of adipocytes on OTSCC proliferation after co-culture, the cells were seeded as above in sextuplicate for each condition. After 72 h of co-culture, the Transwells were discarded, and OTSCC cells from the six wells were detached and combined. Next, 5 × 10^3^ OTSCC cells per well were re-seeded on a 96-well plate. The cell proliferation was estimated with the IncuCyte S3 Live Cell Analysis system (Sartorius) proliferation module for a total of 72 h (10x objective, 2 images per well every 6 h).

### Scratch wound healing assay

To analyse horizontal migration, 3 × 10^4^ OTSCC (co-cultured with adipocytes as above) per well were re-seeded on a 96-well ImageLock™ plate (Sartorius) in sextuplicate for each condition. After 24 h, the scratch wounds were created with a WoundMaker™ device (Sartorius). Horizontal migration was assessed with the IncuCyte scratch wound assay module (10x objective, 1 image per well, imaged every 3 h for 9 h) in an IncuCyte S3 Live Cell Analysis system (Sartorius). This experiment was independently repeated three times.

To evaluate the effects of EVs on OTSCC cell horizontal invasion, we coated the wells of a 96-well ImageLock™ plate (Sartorius) with 50 µl of 300 µg/ml Myogel, a matrix derived from human myoma tissue and left overnight in the cell culture incubator. On the next day, excess Myogel was removed, and 3 × 10^4^ HSC-3 and SCC-25 cells were seeded in each well and left to adhere overnight. Homogeneous scratch wounds were created using a WoundMaker™ device (Sartorius). The wells were washed two times with medium and then 50 µl of Myogel-collagen matrix (2.4 mg/ml Myogel and 0.8 mg/ml type I rat tail collagen) (Corning) was added on top of the cells. EVs isolated from differentiated adipocytes were added to the wells (9 × 10^8^ vesicles/well) with complete media to a total of 400 µl. Horizontal invasion was assessed with the NIS Elements-F program (4x objective, 1 image per well, imaged every 6 h for 12 h). The experiment was repeated independently three times.

### Transwell migration assay

2.2 × 10^4^ differentiated adipocytes per well or adipocyte culture media as a control were plated on a 24-well plate. 7 × 10^4^ OTSCC cells were added per Transwell insert (Corning) with 8 μm pore size. After 24 h, 48 h, and/or 72 h, the cells were fixed with 4% paraformaldehyde for 15 min RT, washed with 1 x PBS, and stained with 1% toluidine blue in 1% sodium tetraborate solution. After washing with dH_2_O, the remaining dye was detached by dipping the insert into 1% SDS. Finally, the absorbance was measured at 650 nm with Victor3V (Perkin Elmer). The experiment was repeated three times.

### Analysis of adipokines

3 × 10^4^ HSC-3 cells per well were plated onto a 24-well plate. 1 × 10^4^ differentiated adipocytes or adipocyte culture media as control were placed into the Transwell chambers with 8 μm pore size (Corning). The cells were co-cultured for 72 h after which the conditioned media were collected from the bottom wells, combined, and clarified by centrifugation (4 min, 394 x g). HSC-3 cells’ whole-cell proteins were extracted into lysis buffer (20 mM Tris-HCl pH 8.0, 137 mM NaCl, 10% glycerol, 2 mM EDTA, 1% nonidet 40, supplemented with approtinin, leupeptin, and pepstatin A (all from Sigma Aldrich), 10 µg/ml each) and incubated at 4°C for 30 min shaking and centrifuged for 5 min at 14 000 x g to pellet the cell debris. The amount of total protein was analysed with DC Protein assay (Bio-Rad). Cell lysates (200 µg) and conditioned media with and without adipocytes in co-culture were examined with Proteome Profiler Human Adipokine Array Kit (R&D Systems) according to the manufacturer’s instructions. All analyses were performed from the pooled samples from six wells. The membranes were visualized with the LAS-3000 imaging system (Fuji), and intensities were measured with Image Studio Lite version 5.2 (LI-COR Biosciences).

### Enzyme-linked immunosorbent assay

Cytokines IL-6 and IL-8 were measured from cell lysates and conditioned medias using human IL-6 Enzyme-linked immunosorbent assay (ELISA) kit (88-7066-88, Invitrogen) and human IL-8 ELISA kit (88-8086-88, Invitrogen) according to the manufacturer’s instructions.

### Statistical methods

Overall survival was estimated with the Kaplan-Meier method and statistical comparison was performed with log-rank test. Correlations between adipocyte and clinical parameters were evaluated with Pearson’s correlation test, Kruskal-Wallis test, Chi-square test, or Fisher’s exact test according to parameter type and normality of distribution. Two-tailed Student’s T-test assuming equal variances were used to evaluate differences in means for measured in vitro assays’ parameters. Statistical analyses were performed with IBM SPSS Statistics for Windows software, version 25 and above (IBM Corp) for analyses related to survival and clinical parameters and with Microsoft Excel for in vitro assays. Results from one representative experiment are shown in cases of repeated experiments. Statistical significance was set at *p* < 0.05.

## Supplementary Information

Below is the link to the electronic supplementary material.

Supplementary Material 1

## Data Availability

All data generated and analysed in this study are available within the paper and its Supplementary Information file.
