# Supplementary material for: Adipocytes enhance tongue cancer progression potentially via IL-6 and extracellular vesicles
Source: Sci Rep. 2025 Nov 21;15:41191. doi: 10.1038/s41598-025-24711-3 (PMC12639075; doi:10.1038/s41598-025-24711-3)
Supplement: Supplementary file 1 — Supplementary Material 1 [file 41598_2025_24711_MOESM1_ESM.pdf]

Adipocytes enhance tongue cancer progression potentially via IL-6 and extracellular vesicles

Supplementary information file: Figures, figure legends, tables and full blot images

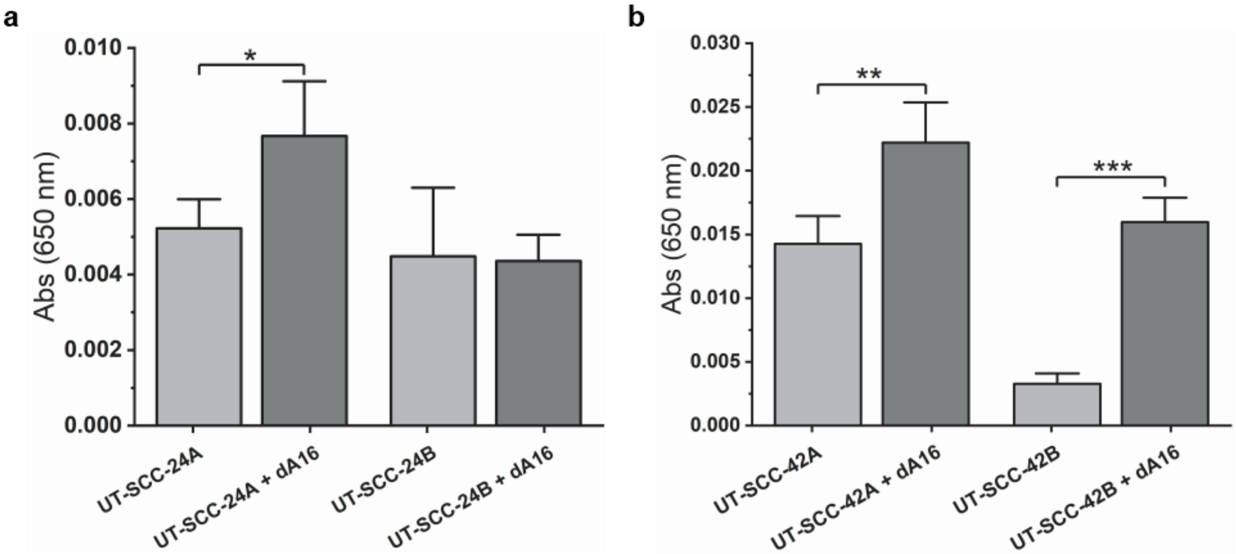

Supplementary Figure 1. Adipocytes increased the migration of UT-SCC24 and UT-SCC42 cell lines. Migration of UT-SCC-24A and UT-SCC-24B **(a)** as well as UT-SCC42A and UT-SCC42B **(b)** cell lines towards differentiated adipocytes (dA16) as measured with Transwell assay. Asterisks indicate statistical significance as evaluated with Student's T-test, *p<0.05, **p<0.01, and ***p<0.001.

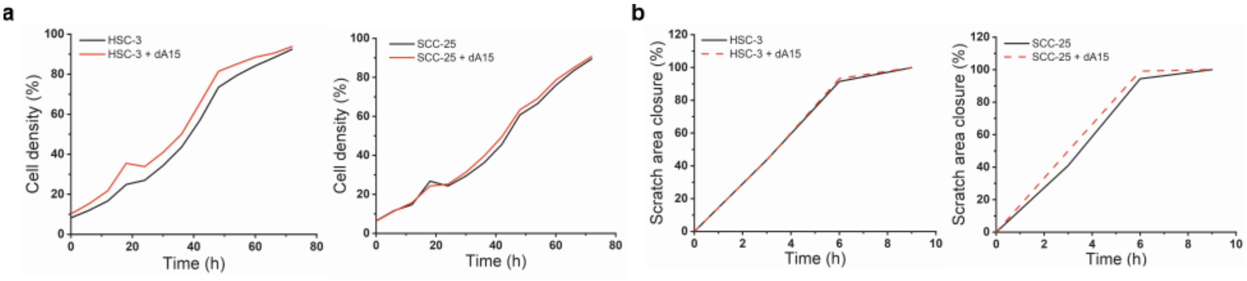

Supplementary Figure 2. Effect of adipocytes on OTSCC cell proliferation and migration requires active interaction. OTSCC cell (HSC-3 and SCC-25) proliferation **(a)** and migration **(b)** after removing the OTSCC cells from co-culture with differentiated adipocytes (dA15) as analysed with Incucyte.

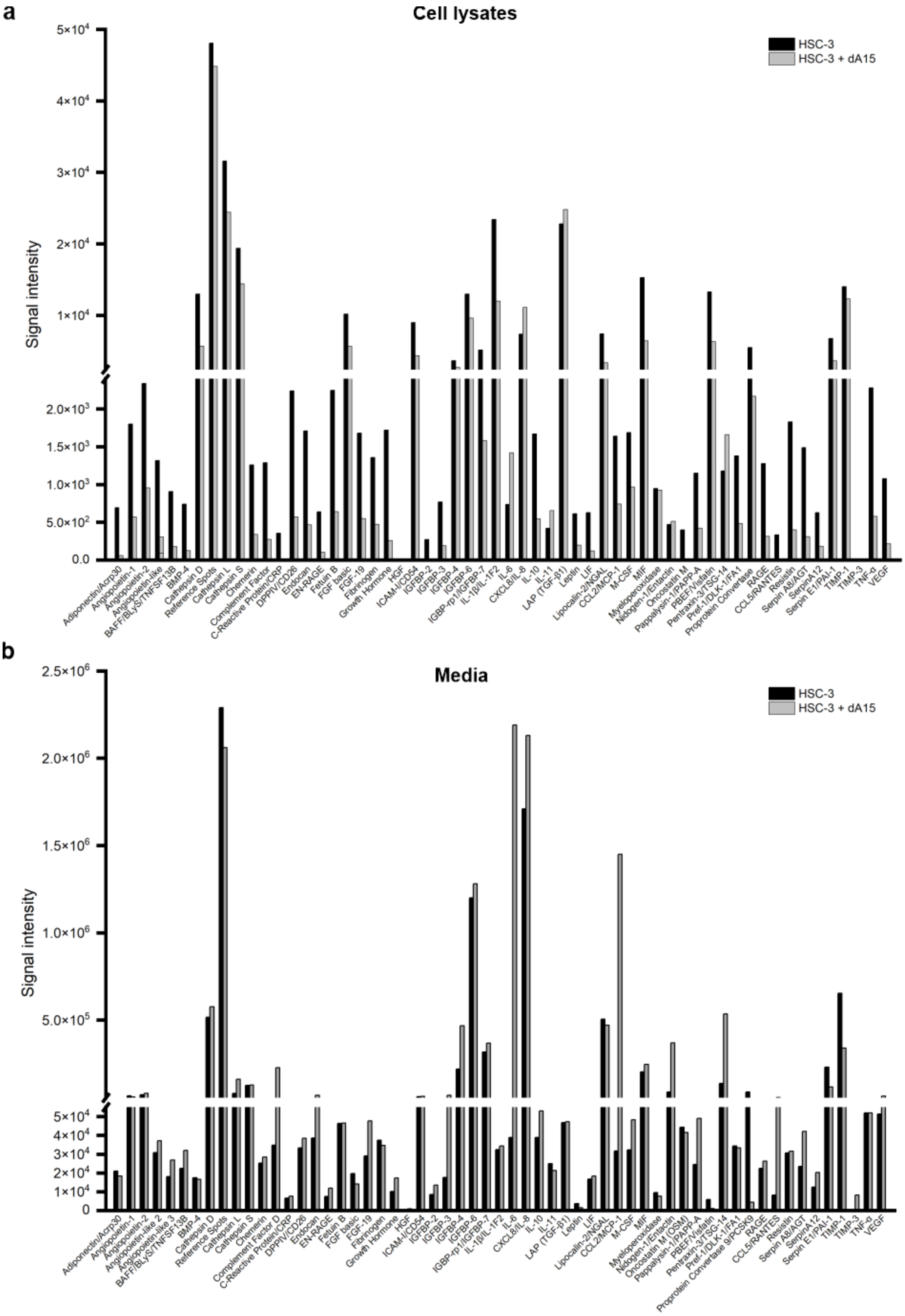
a
Cell lysates
HSC-3
HSC-3 + dA15
Signal intensity
5×10⁴
4×10⁴
3×10⁴
2×10⁴
1×10⁴
2.0×10³
1.5×10³
1.0×10³
5.0×10²
0.0
Adiponectin/Acrp30
Angiopoietin-1
Angiopoietin-2
Angiopoietin-like
BAFF/BLyS/TNFSF13B
BMP-4
Cathepsin D
Reference Spots
Cathepsin L
Cathepsin S
Chemerin
Complement Factor
C-Reactive Protein/CRP
DPPIV/CD26
Endocan
EN-RAGE
Fetuin B
FGF basic
FGF-19
Fibrinogen
Growth Hormone
HGF
ICAM-I/CD54
IGFBP-2
IGFBP-3
IGFBP-4
IGFBP-6
IGBP-rp1/IGFBP-7
IL-1β/IL-1F2
IL-6
CXCL8/IL-8
IL-10
IL-11
LAP (TGF-β1)
Leptin
LIF
Lipocalin-2/NGAL
CCL2/MCP-1
M-CSF
MIF
Myeloperoxidase
Nidogen-1/Entactin
Oncostatin M
Pappalysin-1/PAPP-A
PBEF/Visfatin
Pentraxin-3/TSG-14
Pref-1/DLK-1/FA1
Proprotein Convertase
RAGE
CCL5/RANTES
Resistin
Serpin A8/AGT
SerpinA12
Serpin E1/PAI-1
TIMP-1
TIMP-3
TNF-α
VEGF
b
Media
HSC-3
HSC-3 + dA15
Signal intensity
2.5×10⁶
2.0×10⁶
1.5×10⁶
1.0×10⁶
5.0×10⁵
5×10⁴
4×10⁴
3×10⁴
2×10⁴
1×10⁴
0
Adiponectin/Acrp30
Angiopoietin-1
Angiopoietin-2
Angiopoietin-like 2
Angiopoietin-like 3
BAFF/BLyS/TNFSF13B
BMP-4
Cathepsin D
Reference Spots
Cathepsin L
Cathepsin S
Chemerin
Complement Factor D
C-Reactive Protein/CRP
DPPIV/CD26
Endocan
EN-RAGE
Fetuin B
FGF basic
FGF-19
Fibrinogen
Growth Hormone
HGF
ICAM-I/CD54
IGFBP-2
IGFBP-3
IGFBP-4
IGFBP-6
IGBP-rp1/IGFBP-7
IL-1β/IL-1F2
IL-6
CXCL8/IL-8
IL-10
IL-11
LAP (TGF-β1)
Leptin
LIF
Lipocalin-2/NGAL
CCL2/MCP-1
M-CSF
MIF
Myeloperoxidase
Nidogen-1/Entactin
Oncostatin M (OSM)
Pappalysin-1/PAPP-A
PBEF/Visfatin
Pentraxin-3/TSG-14
Pref-1/DLK-1/FA1
Proprotein Convertase 9/PCSK9
RAGE
CCL5/RANTES
Resistin
Serpin A8/AGT
SerpinA12
Serpin E1/PAI-1
TIMP-1
TIMP-3
TNF-α
VEGF

Supplementary Figure 3. OTSCC cell lysates and media from co-cultures with adipocytes showed increased levels of various cytokines. Proteome Profiler Human Adipokine Array Kit was used to detect various cytokines from cell lysates **(a)** and secretome (media) **(b)** from HSC-3 cells co-cultured with and without differentiated adipocytes (dA15).

Supplementary Table 1. Patient characteristics.

Patient characteristics		Amount of adipocytes		Inflammation		Distance between cancer cells and adipocytes		All patients (n=65)
		0-50%	50-100%	Yes	No	0 µm	> 0 µm	
		n (%)	n (%)	n (%)	n (%)	n (%)	n (%)	n (%)
**Sex**	Female	11 (35.5)	17 (50)	23 (42.6)	5 (45.5)	21 (42.9)	7 (43.8)	28 (43.1)
	Male	20 (64.5)	17 (50)	31 (57.4)	6 (54.5)	28 (57.1)	9 (56.3)	37 (56.9)
**Age**	<50y	6 (19.4)	5 (14.7)	10 (18.5)	1 (9.1)	7 (14.3)	4 (25)	11 (16.9)
	50-69y	13 (41.9)	14 (41.2)	21 (38.9)	6 (54.5)	18 (36.7)	9 (56.3)	27 (41.5)
	≥70y	12 (38.7)	15 (44.1)	23 (42.6)	4 (36.4)	24 (49.0)	3 (18.8)	27 (41.5)
**Amount of adipocytes**	0-50%			21 (38.9)	10 (90.9)	19 (38.8)	12 (75.0)	31 (47.7)
	50-100%			33 (61.1)	1 (9.1)	30 (61.2)	4 (25.0)	34 (52.3)
**Distance**	0 µm	19 (61.3)	30 (88.2)	43 (79.6)	6 (54.5)			49 (75.4)
	> 0 µm	12 (38.7)	4 (25.0)	11 (20.4)	5 (45.5)			16 (24.6)
**Inflammation**	Little/no	1 (4.5)	10 (23.3)			6 (12.2)	5 (31.3)	11 (16.9)
	Average	16 (72.7)	17 (39.5)			24 (49)	9 (56.3)	33 (50.8)
	High	5 (22.7)	16 (37.2)			19 (38.8)	2 (12.5)	21 (32.3)
**pT**	1-2	17 (89.5)	25 (83.3)	37 (86.0)	5 (83.3)	34 (81)	8 (88.9)	42 (85.7)
	3-4	2 (10.5)	5 (16.7)	6 (14.0)	1 (16.7)	8 (19)	1 (11.1)	7 (14.3)
**pN**	0	6 (85.7)	17 (81)	21 (80.8)	2 (100)	19 (86.4)	4 (66.7)	23 (82.1)
	1-2	1 (14.3)	4 (19)	5 (19.2)	0	3 (13.6)	2 (33.3)	5 (17.9)
**cM**	0	25 (92.6)	29 (100)	49 (100)	7 (77.8)	44 (95.7)	12 (100)	56 (96.6)
	1	2 (7.4)	0	0	2 (22.2)	2 (4.3)	0	2 (3.4)
**Grade**	Unknown							12 (18.5)
	1	7 (35)	12 (36.4)	17 (37)	2 (28.6)	14 (33.4)	5 (45.5)	19 (29.2)
	2	10 (50)	16 (48.5)	21 (45.6)	5 (71.4)	20 (47.6)	6 (54.5)	26 (40)
	3	3 (15)	5 (15.1)	8 (17.4)	0	8 (19.0)	0	8 (12.3)
**Status**	Alive	19 (61.3)	26 (76.5)	41 (75.9)	4 (36.4)	33 (67.3)	12 (75)	45 (69.2)
	Dead	12 (38.7)	8 (23.5)	13 (24.1)	7 (63.6)	16 (32.7)	4 (25)	20 (30.8)
**Recurrence**	No	23 (74.2)	27 (79.4)	43 (78.2)	7 (70)	38 (77.6)	12 (75)	50 (76.9)
	Yes	8 (25.8)	7 (20.6)	12 (21.8)	3 (30)	11 (22.4)	4 (25)	15 (23.1)
**Sample type**	Resection	14 (56)	31 (93.9)	40 (83.3)	5 (50)	38 (82.6)	7 (58.3)	45 (77.6)
	Other	11 (44)	2 (6.1)	8 (16.7)	5 (50)	8 (17.4)	5 (41.7)	13 (22.4)
**Alcohol**	Unknown							41 (63.1)
	No	2 (20)	3 (21,4)	4 (20)	1 (25)	4 (23.5)	1 (14.3)	5 (7.7)
	Yes	8 (80)	11 (78,6)	16 (80)	3 (75)	13 (76.5)	6 (85.7)	19 (29.2)
**Tobacco**	Unknown							26 (40)
	No	4 (25)	6 (26,1)	8 (22.9)	2 (28.6)	7 (23.3)	3 (33.3)	10 (15.4)
	Yes	12 (75)	17 (73,9)	27 (77.1)	5 (71.4)	23 (76.7)	6 (66.7)	29 (44.6)

cM=clinical status of distant metastasis, pN=pathological node status, pT=pathological tumour grade

Full western plot images

E-cadherin - OTSCC cells co-cultured with adipose, myoma or no tissue (Figure 2D)

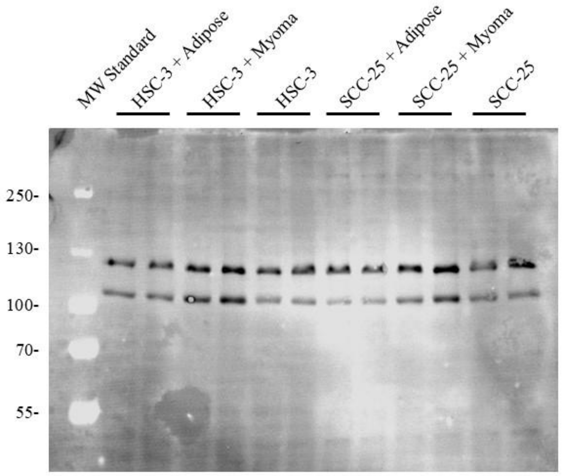

E-cadherin (#3195, Cell Signaling Technology)

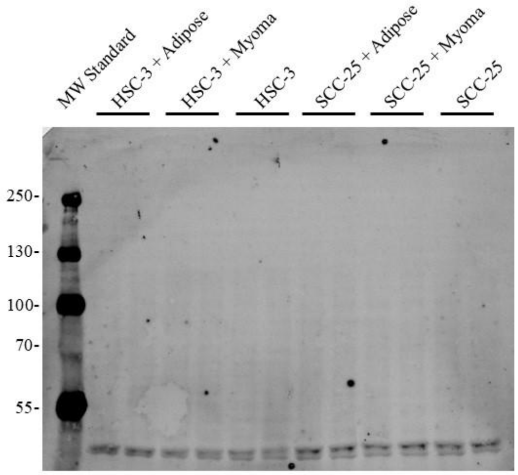

B-actin (ab8226, Abcam)

E-cadherin - OTSCC cells co-cultured with or without differentiated adipocytes (dA15) (Figure 3C)

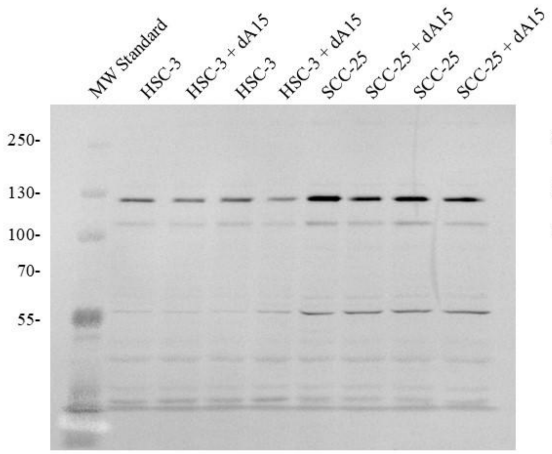

E-cadherin (#3195, Cell Signaling Technology)

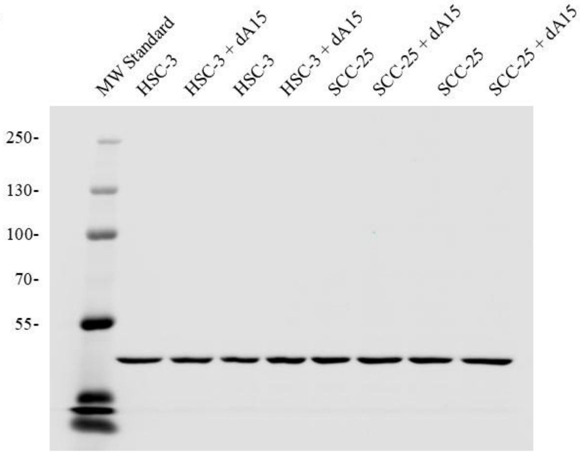

B-actin (ab8226, Abcam)

Vimentin - OTSCC cells co-cultured with or without differentiated adipocytes (dA15) (Figure 3E)

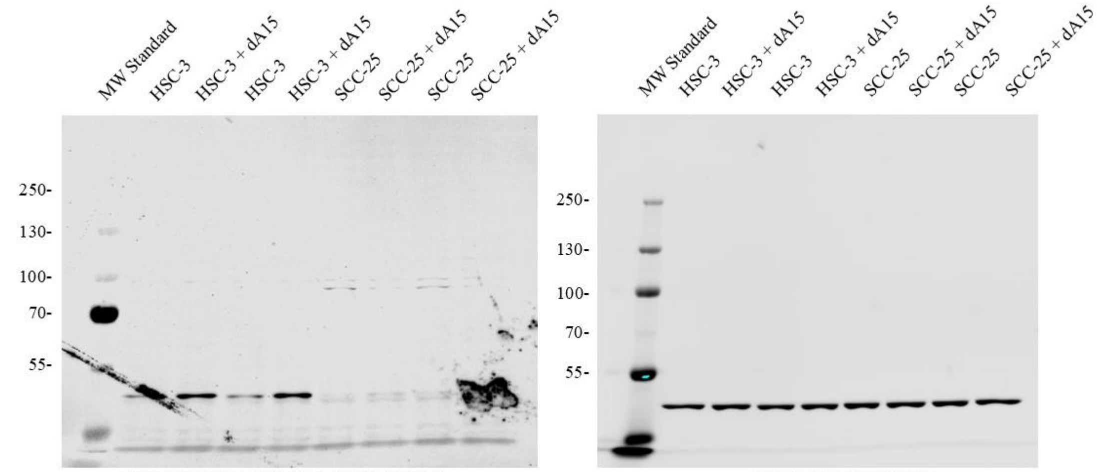

Vimentin (NBPI 19480, Novus Biologicals) B-actin (ab8226, Abcam)
